# Supportive Relationships Mitigate the Effect of Cumulative Exposure to Adverse Childhood Experiences on Depression, Anxiety, Stress, and Suicide Considerations—The Arizona Youth Risk Behavior Survey

**DOI:** 10.3390/children11020161

**Published:** 2024-01-26

**Authors:** Bin C. Suh, M. Shayne Gallaway, Martin F. Celaya

**Affiliations:** 1 Arizona Department of Health Services, Phoenix, AZ 85007, USA; martin.celaya@azdhs.gov; 2 United States Public Health Service, Rockville, MD 20852, USA; michael.s.gallaway.mil@health.mil

**Keywords:** mental health, COVID-19, adolescents, interpersonal support, Adverse Childhood Experiences (ACEs), Positive Childhood Experiences (PCEs)

## Abstract

Declining adolescent mental health is a significant public health concern during the COVID-19 pandemic. Social distancing and stay-at-home orders have led to missed social connections with peers and adults outside households, and this has increased the risk of mental health problems in children and adolescents, particularly those with adverse childhood experiences (ACEs). Studies have shown that strong interpersonal support improves adolescent mental health. We examined the association between ACEs and poor mental health (including stress, anxiety, and depression) and how the presence of interpersonal support from caring adults and friends and school connectedness can mitigate this relationship among adolescents in Arizona. This study analyzed data from the 2021 Arizona Youth Risk Behavior Survey (YRBS; *n* = 1181), a population-based survey conducted biennially across the United States. The Arizona sample included high school students in grades 9–12 who were enrolled in public and charter schools. This study revealed that nearly three of four adolescents experienced an ACE, and one of five experienced ≥4 ACEs. Compared with adolescents who experienced zero ACEs, those with ≥4 ACEs experienced less interpersonal support from caring adults, friends, and school and more frequently reported poor mental health and suicidal thoughts. However, adolescents with interpersonal support consistently reported lower rates of mental health issues, even with exposure to multiple ACEs. Post-pandemic programs to improve social relationships with adults, peers, and schools are critical, especially for adolescents with multiple adversities.

## 1. Introduction

The COVID-19 pandemic has had a significant effect on the mental health of adolescents. A recent study showed adolescents experienced increased stress and uncertainty; social distancing and stay-at-home orders led to missed social connections with peers and adults outside their households, leading to a decline in overall wellbeing and an increase in mental health issues [1]. According to the Centers for Disease Control and Prevention (CDC), an estimated one out of five children will have experienced a mental disorder in 2022, with an estimated annual cost of $247 billion for treatment and management of childhood mental disorders [2]. In another study, the CDC found two in five adolescents reported experiencing poor mental health during the pandemic; 20% of adolescents considered committing suicide, while 9% had attempted it in the preceding year [3]. These findings highlight the importance of examining suicide considerations as a specific aspect of adolescent mental health in the context of the COVID-19 pandemic.

Declining adolescent mental health has become a significant public health concern, particularly for those with adverse childhood experiences (ACEs) [4]. ACEs refer to potentially traumatic events that occur in childhood, such as abuse and neglect, and household dysfunction, including parental divorce/separation or living with someone with a mental illness [5]. In the 1990s, in a groundbreaking study on ACEs, Felitti and his colleagues found associations between cumulative exposure to ACEs and various health complications (e.g., depression, suicide attempt, cancer, chronic lung disease) among about 17,000 adult members of the Kaiser Permanente health maintenance organization in Southern California [6]. Since then, decades of research have shown a consistent dose–response relationship between ACEs and chronic diseases, injuries, and death [7]. This relationship can be explained by the body’s response to chronic exposure to stress, or *toxic stress*, due to childhood adversity [8]. Shonkoff and colleagues [8] found that toxic stress triggers the release of high levels of stress hormones such as cortisol and adrenaline, which can damage various systems in the body when consistently elevated; additionally, toxic stress can overload an individual’s coping mechanisms and make them more susceptible to conditions such as anxiety, depression, and post-traumatic stress disorder. As a result, multiple ACEs without proper intervention to mitigate their harmful impact on health could lead to a shorter life expectancy [7,8].

## 2. The Role of Protective Factors in Mitigating the Impact of Adverse Childhood Experiences (ACEs)

Notably, there has been a new paradigm shift in the ACE literature that emphasizes the role of safe, supportive, and nurturing relationships (SSNR), which are conceptualized as Positive Childhood Experiences (PCEs), in preventing or mitigating ACEs [9,10]. This changing viewpoint emphasizes the significant impact of positive interactions on breaking the intergenerational cycle of maltreatment and developing resilience in individuals [11]. Conceptually opposite to ACEs, PCEs are experiences during childhood that foster emotional wellbeing and strengthen a person’s ability to navigate challenges successfully [12]. Major categories of PCEs identified by the literature include (1) after-school activities, (2) volunteering in a community, school, or church, (3) mentoring for advice or guidance, (4) sharing ideas with caregivers, (5) living in safe neighborhoods, (6) living in supportive neighborhoods, and (7) family resilience. Studies have shown that interpersonal solid support from parents or peers can enhance the mental health of adolescents [13] and that SSNRs decrease the odds of risky behavior, particularly among individuals who have experienced multiple ACEs [14]. However, adolescents with multiple ACEs may often have less access to interpersonal support. Childhood adversity often co-occurs with disrupted relationships with adults, friends, and within the community; thus, individuals and families with ACEs may have fewer opportunities to build strong emotional support and connections.

## 3. Use of Youth-Reported Data for ACE and PCE Research

Many studies on ACEs and PCEs have utilized data from adults who retrospectively report their childhood adversity (e.g., Behavioral Risk Factor Surveillance System) [15,16]. Alternatively, parents or guardians have reported on behalf of children aged 0–17 (e.g., National Survey of Children’s Health) [17]. However, using different types of data, such as youth-based data that are reported directly from youth regarding their experiences of adversity and protective factors, is also essential to triangulate childhood adversity. The Youth Risk Behavior Survey (YRBS) has incorporated 12 questionnaires on ACEs, spanning from physical abuse and neglect to community violence and discrimination, and three questions on PCEs (i.e., having a caring adult and friend and feeling connected to people at school) to examine the prevalence of such experiences among youth enrolled in public and charter schools throughout the United States [18]. Even though not all states have incorporated the questionnaires, several states, including Arizona, utilize the YRBS data on ACEs and PCEs for public health surveillance. Several studies have used the YRBS data to report the prevalence and impact of ACEs and PCEs on health behavior [19,20]. However, to the authors’ knowledge, there is limited literature available that has explored the prevalence of youth-reported SSNRs with individuals in adolescents’ close proximity, such as close adults, friends, and people at school, and their role in reducing the impact of ACEs on the mental health of adolescents in the era of COVID-19.

## 4. Purpose

The purpose of this cross-sectional study is to (1) examine the association between ACEs and self-reported mental health (during the COVID-19 pandemic and in the past 30 days) and suicide considerations in the past 12 months and (2) investigate how the presence of interpersonal support from caring adults and friends and school connectedness can mitigate this association among adolescents in Arizona. This study aimed to gain an understanding of the protective role of PCEs in the form of interpersonal support from adolescents’ proximal relationships (e.g., family, friends, and school) in lowering the harmful impact of ACEs.

## 5. Materials and Methods

The 2021 Arizona Youth Risk Behavior Survey (YRBS) data was utilized to gain direct insights from youth in public schools. The YRBS is a biennial, population-based survey of high school students in grades 9–12, conducted in Arizona in partnership with the CDC, to collect information on a wide range of health behaviors, such as vaping, tobacco use, and self-reported mental health status, as well as risk and protective factors [21,22]. In partnership with the CDC, the YRBS uses a multistage cluster design by selecting primary sampling units, schools, and classes to obtain a representative sample of students in grades 9–12 who attend public schools. As for the YRBS sampling strategy, public schools with any of grades 9–12 were sampled with probability proportional to school enrollment size, and then intact classes from either a required subject (e.g., English or social studies) or a required period (e.g., homeroom or second period) were sampled randomly. All students in selected classes who could independently complete the survey within a single class period were eligible to participate, and student participation was anonymous and voluntary. The protocol for the YRBS was approved by the institutional review boards at the CDC and ICF, the survey contractor. Data collection adhered to applicable federal law and CDC policy. The 2021 survey in Arizona required passive parental consent, where parents were informed about the survey prior to survey administration and signed and returned a form only if they refused to allow their child to participate. The YRBS data are weighted based on sex, race/ethnicity, and grade to adjust for school and student nonresponse and to make the data representative of the population of Arizona students in grades 9–12. More information about the methodology of the 2021 YRBS has been reported elsewhere [23]. This study’s analysis was conducted using data from the 2021 Arizona YRBS cycle. About 79% of eligible sampled students and 58% of eligible schools in Arizona participated in the survey. The overall response rate for the survey, encompassing both, was 46%.

## 6. Measures

### 6.1. Adverse Childhood Experiences (ACEs)

In the 2021 YRBS, 12 questions asking ten types of lifetime exposure to childhood adversity were utilized for this study, including physical/sexual/emotional abuse, racial/sexual discrimination, household alcohol/drug abuse, household mental illness, household incarceration, and witnessing community and domestic violence (Appendix A shows the ACEs questionnaires along with the thresholds used to identify positive ACE cases). All ACE measures were dichotomized as yes (i.e., 1) versus no (i.e., 0). The cumulative number of lifetime types of ACEs was calculated and categorized as 0, 1, 2–3, and ≥4 ACEs, consistent with how previous ACEs research categorized cumulative ACE scores [24,25].

### 6.2. Interpersonal Support

The data also included three questions about positive experiences salient in adolescent development, referred to as “the dimensions of interpersonal support”. Using a five-point Likert scale to answer questions, adolescents were asked, “During your life, how often have you felt that you were able to talk to an adult in your family or another caring adult about your feelings?” Responses to this question ranged from “never” to “always” and were then coded into two mutually exclusive categories (e.g., “always/most of the time” = 1 and “never/rarely/sometimes” = 0), as suggested by the codebook, in order to assess the presence of caring adults. Similarly, a question about supportive friends asked adolescents, “During your life, how often have you felt that you were able to talk to a friend about your feelings?” Responses to this question ranged from “never” to “always” and were then coded into two categories (e.g., “always/most of the time” = 1 and “never/rarely/sometimes” = 0), as suggested by the codebook. As for school connectedness, adolescents were asked, “Do you agree or disagree that you feel close to people at your school?” on a five-point Likert scale ranging from “strongly disagree” to “strongly agree.” Responses were then dichotomized (e.g., “strongly agree/agree” = 1 and “not sure/disagree/strongly disagree” = 0), as suggested by the codebook.

### 6.3. Poor Mental Health and Suicide Considerations

The 2021 YRBS also included questions to examine poor mental health during the COVID-19 pandemic and in the past 30 days, as well as suicide considerations in the past 12 months. For the question about self-reported mental health status during the pandemic, participants were asked, “During the COVID-19 pandemic, how often was your mental health not good? (Poor mental health includes stress, anxiety, and depression.)” Response options ranged from “never” to “always.” “Most of the time” and “always” were grouped and considered poor mental health. The question about self-reported mental health status in the past 30 days asked, “In the past 30 days, how often was your mental health not good?” Similarly, response options ranged from “never” to “always”, and “most of the time” and “always” were considered poor mental health. Lastly, the question about suicide considerations asked, “During the past 12 months, did you ever seriously consider attempting suicide?” The response options were “yes” and “no”.

## 7. Data Analysis

Descriptive analytical techniques were used to produce frequencies and proportions. Chi-square tests were used to measure the association between ACEs, interpersonal support, poor mental health, and suicide considerations. A *p*-value of less than 0.05 was considered statistically significant. SAS (version 9.4; SAS Institute, Cary, NC, USA) was used to conduct all analyses, and appropriate procedures were used to account for the YRBS complex sampling methods.

## 8. Results

### Participants

The total number of participants was 1181. The demographic characteristics of adolescents who participated in the study are shown in Table 1. Twenty percent of the participants were 14 years old or younger, 24% were 15 years old, 25% were 16 years old, and 32% were 17 years old or older. About a quarter of the participants were from 9th to 12th grade. Additionally, around 51% of the participants were female, and the largest race/ethnicity group was Hispanic, accounting for 46% of all participants. Non-Hispanic White adolescents were the second-largest group, representing 39% of the participants.

Seventy-seven percent of adolescents experienced at least 1 ACE, and 22% reported ≥4 ACEs during their lifetime (Table 1). The most commonly reported ACEs were emotional abuse (59%), household mental illness (43%), and household drug abuse (37%). Nearly 2 of 5 adolescents reported their mental health was not good during COVID-19 or during the past 30 days, and 24% reported seriously considering attempting suicide in the past 12 months (as shown in Table 1).

Demographic differences were also noted. Females reported higher ACE cumulative scores than males, as 36% of females reported 4+ ACEs or 2–3 ACEs, compared with 15% and 33% of males, respectively (X^2^ [3, N = 11,174] = 42.0, *p* < 0.0001). Similarly, compared with males, females were more likely to report poor mental health during COVID-19 (54% vs. 25%; X^2^ [1, N = 1108] = 98.7, *p* < 0.0001) and in the past 30 days (51% vs. 22%; X^2^ [1, N = 1110] = 101.7, *p* < 0.0001). Consistently, females were more likely to report suicide considerations than males (32% vs. 14%; X^2^ [1, N = 1153] = 53.5, *p* < 0.0001). As for interpersonal support, school connectedness was notably different by sex, as more males reported school connectedness than females (50% vs. 42%; X^2^ [1, N = 1108] = 7.5, *p* < 0.05). Non-Hispanic White adolescents were more likely to report caring adults (45%), compared with Hispanic (34%) and non-Hispanic multiracial/others (38%; X^2^ [2, N = 1106] = 13.9, *p* < 0.01). Similarly, non-Hispanic White adolescents were more likely to have supportive friends (53%), compared with Hispanic (38%) and non-Hispanic multiracial/others (43%; X^2^ [2, N = 1101] = 19.3, *p* < 0.001).

**Figure 1:**
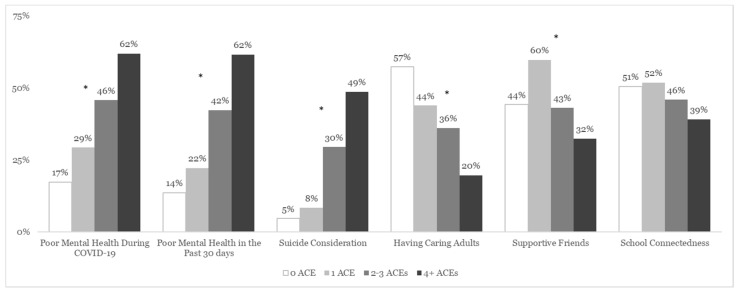
The weighted prevalence of poor mental health (MH) during COVID-19 and past 30 days, suicide considerations, interpersonal support, and school connectedness among adolescents, by ACE score–Youth Risk Behavior Survey (YRBS), Arizona, 2021 (N = 1181). Note. * *p* < 0.5; ACEs = Adverse Childhood Experiences.

The prevalence of poor mental health during COVID-19 and suicide considerations increased with an increasing number of ACEs (see Figure 1). Adolescents with higher ACE scores were more likely to report poor mental health during COVID-19 (X^2^ [3, N = 1115] = 121.0, *p* < 0.0001), in the past 30 days (X^2^ [3, N = 1117] = 151.0, *p* < 0.001), and suicide considerations in the past 12 months (X^2^ [3, N = 1160] = 180.4, *p* < 0.0001). Conversely, adolescents with multiple ACEs reported less interpersonal support than adolescents with 0 ACEs (except for school connectedness (X^2^ [3, N = 1115] = 9.5, *p* = 0.08)); fewer reported caring adults (X^2^ [3, N = 1119] = 79.3, *p* < 0.0001) or supportive friends (X^2^ [3, N = 1114] = 35.3, *p* < 0.001).

**Figure 2:**
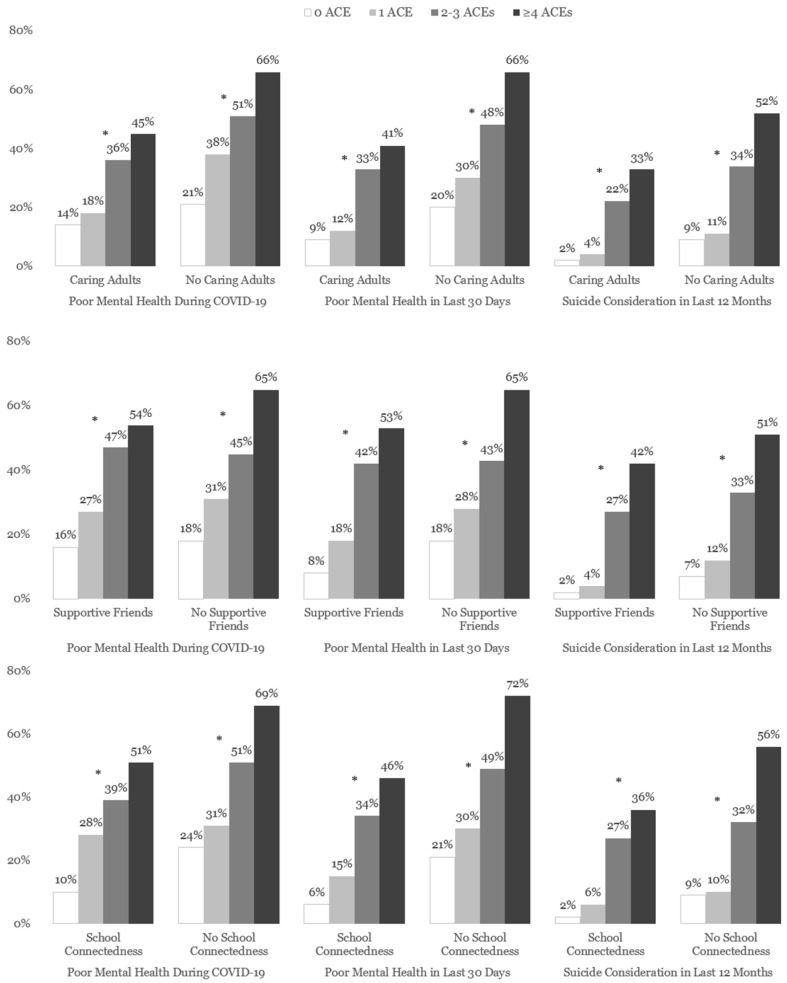
The weighted prevalence of poor mental health (MH) during COVID-19 and past 30 days, suicide considerations, interpersonal support, and school connectedness among adolescents, by ACE score and interpersonal support type–Youth Risk Behavior Survey (YRBS), Arizona, 2021 (N = 1181). Note. * *p* < 0.5; ACEs = Adverse Childhood Experiences.

The significant dose-response relationships between ACEs and poor mental health and suicide considerations remain consistent, regardless of the presence of interpersonal support (Figure 2). However, the prevalence of poor mental health during COVID-19 and in the past 30 days and suicide considerations in the past 12 months was also lower among adolescents who had interpersonal support, regardless of ACE scores. These differences were more visible among those with ≥4 ACEs. Among adolescents who had ≥4 ACEs, the prevalence of poor mental health during COVID-19 (45% vs. 66%; X^2^ [1, N = 1106] = 6.9, *p* < 0.01), poor mental health in the past 30 days (41% vs. 66%; X^2^ [1, N = 1105] = 10.2, *p* < 0.01), and suicide considerations (33% vs 52%; X^2^ [1, N = 1105] = 5.7, *p* < 0.01) were all significantly lower among those who had caring adults compared with those who did not have caring adults. Consistently, among adolescents with ≥4 ACEs, the prevalence of poor mental health during COVID-19 (54% vs. 65%; X^2^ [1, N = 1103] = 2.7, *p* = 0.12), poor mental health in the past 30 days (53% vs. 65%; X^2^ [1, N = 1100] = 3.1, *p* = 0.15), and suicide considerations (42% vs 51%; X^2^ [1, N = 1098] = 1.8, *p* = 0.20) appeared to be lower among those with supportive friends compared with those without supportive friends, although the differences were not statistically significant. Similarly, among adolescents who had ≥4 ACEs, the prevalence of poor mental health during the past 30 days (46% vs. 72%; X^2^ [1, N = 1101] = 15.9, *p* < 0.05), poor mental health during COVID-19 (51% vs 69%; X^2^ [1, N = 1106] = 7.3, *p* = 0.06), and suicide considerations (36% vs 56%; X^2^ [1, N = 1102] = 9.3, *p* < 0.01) was lower among those with school connectedness compared with those without school connectedness.

## 9. Discussion

A substantial number of adolescents in Arizona have experienced at least one ACE, and a fifth have experienced ≥4 ACEs (21%). These findings were fairly consistent with data for adult populations, showing 62% had at least one ACE, and 25% had three or more [26]. This study shows a clear dose-response association between ACEs and poor mental health and suicide considerations. However, adolescents with interpersonal support from adults, friends, or at school tend to have lower rates of poor mental health and suicide considerations, even if they have a history of multiple ACEs. Unfortunately, adolescents with multiple ACEs were less likely to have interpersonal support, highlighting their increased vulnerability to mental health problems [27]. This is concerning because an increasing number of ACEs is associated with many chronic health problems, mental illness, substance misuse, and socioeconomic challenges in adulthood [15,27].

## 10. Poor Mental Health as an Emerging Public Health Issue in Adolescents and the Protective Role of PCEs

What is also striking in the findings is that about 40 percent of adolescents reported poor mental health during the pandemic and in the past 30 days. Several reviews of studies noted the significant impact of the pandemic and the containment measures (e.g., social distancing) on the mental health of children and adolescents, even though these populations make up a small percentage of individuals affected by the disease [28,29]. The extended periods of lockdowns and restrictions caused a disruption in the regular routines and social interactions of adolescents, leading to feelings of isolation and loneliness. The closure of schools meant a loss of in-person social support and educational structure, which had a significant impact on the emotional and developmental wellbeing of these individuals. Furthermore, the unpredictability surrounding the pandemic, along with concerns about personal and family health, exacerbated stress levels among adolescents [28]. Moreover, it is essential to recognize that declining psychological wellbeing among adolescents is a multifaceted issue, which can be exacerbated by factors such as childhood trauma, academic competition, and excessive pressure to succeed academically [30], as well as the pervasive influence of social media [31]. Recognizing these complexities is imperative for developing effective strategies that are specifically tailored to adolescents’ distinctive needs, highlighting the importance of a holistic and tailored response to safeguard the psychological wellbeing of adolescents.

On a positive note, the study’s findings indicate that having PCEs can protect against mental health issues in the population. Adolescents who received SSNRs had lower rates of self-reported poor mental health and suicide considerations, regardless of their ACE scores, including the “no ACE” group. This is consistent with the literature, as the protective role of supportive relationships is well documented by past studies [29]. Recent studies found that, even amid adversity, nurturing and supportive relationships with individuals in close proximity (such as family resilience and connection) were linked with positive health indicators among children [16,17,32]. Regarding the operation of PCEs in one’s development, there are ongoing discussions in the literature on whether PCEs have promotive (direct impact on health) versus protective functions (protective impact on health amid adversity). A recent review of the studies conducted by Han and colleagues [16] highlighted that PCEs may have a more direct impact on mental and physical outcomes rather than moderating the impact of childhood adversity on outcomes. Our study also shows that, even among adolescents with no ACEs, the percentages who reported mental health issues were lower when they had PCEs. Simultaneously, mental health issues were consistently less frequently reported among those with ACEs when they had interpersonal support, suggesting that PCEs may have both promotive and protective functions. With this perspective, the CDC’s Essentials for Childhood framework also underpins the importance of SSNRs [33], along with evidence-based recommendations for pediatrics to address social and emotional wellbeing in the population [34]. Targeted interventions aimed at fostering SSNRs in the daily lives of adolescents serve as a pivotal strategy in promoting the mental wellbeing of adolescents, even in the face of adverse experiences. Furthermore, additional research is essential to identify clear developmental pathways leading to positive health outcomes.

## 11. Implications for Policy and Practice

Addressing the detrimental effects of ACEs and offering support to vulnerable families facing mental health challenges has underscored the imperative for evidence-based public health initiatives; examples include after-school activities, home visitation [35], and mentoring programs [36]. The negative impact of ACEs extends beyond children and adolescents to affect the mental wellbeing of parents and caregivers [36]. This is exacerbated by the pandemic’s social mitigation measures, which have restricted access to crucial resources for families [1]. (Of note, 73% of adolescents have encountered adverse family conditions, such as family violence, during this challenging period [4].) Consequently, implementing holistic yet SSNR-focused programs and support systems within families, schools, and communities is paramount in mitigating these challenges. Rather than solely emphasizing individual responsibility, more public health policies could consider the importance and prioritization of collectively building a strong sense of community. This can be achieved by creating safe and stable communities and enhancing the relationships between adolescents and supportive adults and friends in their environment as they navigate their growth and development [5].

Furthermore, the National Academy of Sciences, Engineering, and Medicine emphasized in its 2019 report, “Vibrant and Healthy Kids: Aligning Science, Practice, and Policy to Advance Health Equity,” that supporting family cohesion and social connections requires systemic changes at the federal, state, and local levels [37] to close policy gaps that cause health inequity in vulnerable populations. Several policy approaches include, but are not limited to, developing and implementing more preventive interventions that are (1) suitable for fathers and other male caregivers, and (2) tailored to meet the needs of vulnerable communities, immigrants, children in foster care, and children with incarcerated parents. Implementing preventive interventions tailored for diverse caregivers and communities is essential to foster SSNRs, particularly for those affected by ACEs.

## 12. Limitations and Strengths

General limitations of the YRBS have been documented previously [38]. In addition, the survey did not fully capture all types, specific details, or the severity and frequency of each childhood adversity. The questionnaire also did not assess the ongoing nature of interpersonal support; instead, it asked whether adolescents had ever received such support in the past. The study’s findings lacked control for demographics when examining the impact of PCEs on the relationship between ACEs and poor mental health. While adjusted odds ratios could be utilized to illustrate changes in the odds of reporting mental health issues by ACE score after accounting for demographic information, future studies with a larger sample size, combining multiyear or cross-state data, would be necessary to obtain stable estimates for the analyses with the inclusion of multiple variables. Instead, the current study focused on subgroup analyses to directly inform ongoing local, county, and state partners on the prevalence of mental health issues by ACE score and the presence of interpersonal support. Notably, the study revealed that the prevalence of poor mental health remained lower even among those reporting no ACEs when caring adults, friends, and school personnel were present; according to the authors’ knowledge, the most effective way to convey these findings was through conducting descriptive group comparisons. Finally, this study does not support any causal relationships among variables used for the analysis, due to the nature of the cross-sectional design. Despite its limitations, the Arizona YRBS is the most extensive survey available for the state’s youth population and shares meaningful insights into the significant impact of ACEs and their support system on the current mental health and wellness of adolescents.

## 13. Conclusions

In conclusion, a significant proportion of adolescents reported ACEs. Adolescents with ≥4 ACEs have less interpersonal support, poorer mental health, and higher suicide risk. Supportive adults, friends, or strong school connections are crucial in mitigating the negative impact of ACEs on mental health. Evidence-based public health initiatives are necessary to address ACEs and support vulnerable families, especially during the COVID-19 pandemic. Public health programs could focus on strengthening connections between adolescents and supportive adults and friends to promote healthy development.

## Figures and Tables

**Table 1:** Sociodemographic and adverse childhood experience (ACE) characteristics of the study population—Youth Risk Behavior Survey (YRBS), Arizona, 2021 (N = 1181).

Characteristic		Weighted %
Age group	14 years old or younger	19.8
	15 years old	23.9
	16 years old	24.5
	17 years old or older	31.9
Grade	9th	25.6
	10th	25.1
	11th	22.7
	12th	25.8
Sex	Male	49.1
	Female	50.9
Race/Ethnicity	Hispanic	45.6
	Non-Hispanic White	38.5
	Non-Hispanic Black	4.6
	Non-Hispanic American Indian Native American	1.4
	Non-Hispanic Asian/Native Hawaiian/Pacific Islander	2.4
	Non-Hispanic Multiracial	7.5
ACEs	Emotional Abuse	59.0
	Household mental illness	42.8
	Household alcohol/drug abuse	37.1
	Witnessed community violence	23.0
	Household incarceration	17.2
	Physical abuse	15.3
	Sexual abuse	15.3
	Racial discrimination	6.5
	Sexual discrimination	6.9
	Witnessed domestic violence	3.3
Co-Occurrence of ACEs	0 ACE	22.9
	1 ACE	19.4
	2–3 ACEs	35.3
	4+ ACEs	22.4
Interpersonal Support	Having caring adults	38.9
	Having supportive friends	44.2
	School connectedness	46.6
Poor Mental Health	Poor mental health during the COVID-19 pandemic	39.2
	Poor mental health in the past 30 days	35.8
Suicide Considerations	Ever seriously considered attempting suicide in the past 12 months	24.1

Note. ACEs = Adverse Childhood Experiences; percentages are weighted estimates based on sex, race/ethnicity, and grade to adjust for school and nonresponse and to make the data representative of Arizona students enrolled in grades 9–12.

## Data Availability

The Arizona YRBS data is available upon request via the Arizona Department of Health Services website (azdhs.gov/yrbs; accessed on 24 January 2024).

## Appendix A

Adverse Childhood Experiences (ACEs) Questionnaires in the 2021 Arizona Youth Risk Behavior Survey (YRBS)

During your life, how often has a parent or other adult in your home sworn at you, insulted you, or put you down?

A. Never
B. Rarely
C. Sometimes
D. Most of the time
E. Always

During the past 12 months, how many times has a parent or other adult in your home sworn at you, insulted you, or put you down?

A. 0 times
B. 1 time
C. 2 or 3 times
D. 4 or 5 times
E. 6 or more times

During your life, how often has a parent or other adult in your home hit, beat, kicked, or physically hurt you in any way?

A. Never
B. Rarely
C. Sometimes
D. Most of the time
E. Always

During the past 12 months, how many times has a parent or other adult in your home hit, beat, kicked, or physically hurt you in any way?

A. 0 times
B. 1 time
C. 2 or 3 times
D. 4 or 5 times
E. 6 or more times

Has an adult or person at least 5 years older than you ever forced you to do sexual things that you did not want to do? (Count such things as kissing, touching, or being physically forced to have sexual intercourse.)

A. Yes
B. No

During your life, how often have your parents or other adults in your home slapped, hit, kicked, punched, or beat each other up?

A. Never
B. Rarely
C. Sometimes
D. Most of the time
E. Always

Have you ever lived with someone who was depressed, mentally ill, or suicidal?

A. Yes
B. No

Have you ever been separated from a parent or guardian because they went to jail, prison, or a detention center?

A. Yes
B. No

Have you ever seen someone get physically attacked, beaten, stabbed, or shot in your neighborhood?

A. Yes
B. No

Have you ever lived with someone who was having a problem with alcohol or drug use?

A. Yes
B. No

During your life, how often have you felt that you were treated badly or unfairly because of your race or ethnicity?

A. Never
B. Rarely
C. Sometimes
D. Most of the time
E. Always

During your life, how often have you felt that you were treated badly or unfairly because of your sexual orientation?

A. Never
B. Rarely
C. Sometimes
D. Most of the time
E. Always
